# Understanding the implementation process of the Adult Day Services Plus program

**DOI:** 10.1186/s12877-025-05757-4

**Published:** 2025-02-13

**Authors:** Quinton D. Cotton, Dionne Bailey, Elle Albers, Steph Ingvalson, Emily Bloomquist, Katie Marx, Keith Anderson, Holly Dabelko-Schoeny, Lauren Parker, Laura N. Gitlin, Joseph E. Gaugler

**Affiliations:** 1 School of Social Work, University of Pittsburgh, Pittsburgh, USA; 2 School of Public Health, University of Minnesota, Minneapolis, USA; 3 Center for Healthy Aging and Innovation, University of Minnesota, Minneapolis, USA; 4 Drexel University College of Nursing and Health Professions, Philadelphia, USA; 5 Johns Hopkins University, Baltimore, USA; 6 University of Mississippi, Oxford, USA; 7 Ohio State University, Columbus, USA; 8 Robert L. Kane Endowed Chair in Long-Term Care & Aging, School of Public Health, University of Minnesota, D351 Mayo (MMC 729), 420 Delaware Street S.E., Minneapolis, MN 55455 USA

**Keywords:** Community-based long-term care, Caregiving, Dementia, Alzheimer’s disease, Long-term care, Implementation science

## Abstract

**Background:**

Among the available evidence-based interventions targeting dementia family caregivers, there is limited evidence on implementation processes that produce desired outcomes (i.e., reductions in depression and burden) for caregivers, people living with dementia (PLWD), and community-based programs themselves. In a national multi-site hybrid effectiveness trial, we investigated the implementation success and challenges of embedding an evidence-based intervention (ADS Plus) targeting dementia family caregivers whose PLWD was enrolled in an adult day service (ADS).

**Methods:**

Informed by the Consolidated Framework for Implementation Research, we conducted a directed qualitative content analysis to understand caregiver (*n* = 15) and staff (*n* = 14) perceptions of facilitators of and potential barriers to implementation of ADS Plus in nine ADS programs to guide future dissemination efforts.

**Results:**

Results demonstrated that successful delivery of ADS Plus was achieved through intervention adaptability, personalization, and structure (innovation); responsiveness of ADS Plus to external changes and intervention marketability (outer domain); presence of aligned goals and familiarity (inner setting); involvement of research staff, connections among practitioners, and meeting caregiver needs (individual domain); and understanding caregivers’ needs and addressing staff capacity to take action (implementation process). This adaptability reassures us of the potential to implement ADS Plus in heterogeneous programmatic settings.

**Conclusion:**

Globally, our results demonstrate that ADS Plus offers a viable community-based solution for supporting dementia family caregivers with high implementation potential for diverse ADS settings.

**Trial registration:**

ClinicalTrials.gov ID: NCT02927821 (Registration Date 10/7/2016).

**Supplementary Information:**

The online version contains supplementary material available at 10.1186/s12877-025-05757-4.

## Background

More than 6 million people are living with dementia (PLWD) in the United States and are supported by over 11 million family and friend caregivers [1, 2]. Decades of research demonstrate unmet needs and a lack of appropriate services for dementia care and management for both PLWD and those who care for them [3–5]. Dementia caregivers report high levels of burden and stress, in part driven by limited access to and availability of evidence-based, dementia-specific supports [4, 6]. Nationally, structural supports for dementia care in many communities exist but are often not readily accessible or adequate due to issues such as service fit, inconvenient location, lack of culturally tailored supports for diverse populations, or an insufficient workforce for aging services [7–9].

Although scientific evidence remains mixed, there now exist several programs that are designed to support the needs of PLWD and their family/friend caregivers (hereafter referred to as “caregivers”) [10–12]. However, the availability of such programs for families living with dementia remains limited, given the number of people living with dementia and their caregivers currently in the U.S. [1]. For example, as of 2016, only 48% of Area Agencies on Aging offered evidence-based caregiver programs (it remains unknown if this percentage has changed since) [13]. Staff training requirements, length, complexity, little alignment with operations and daily workflow, and lack of incentivization/reimbursement are often reasons cited for why community-based providers do not adopt dementia care interventions into their routine programming [11, 12, 14].

There also exist multiple barriers to using community-based services (evidence-based or not) among dementia caregivers. For example, caregivers report having little or no knowledge of available services [15–17]. Furthermore, caregivers tend to report dissatisfaction and challenges when seeking and utilizing services, as many of the supports available do not match their needs [17, 18]. Additionally, the fragmentation of dementia care and support services makes it difficult for caregivers to connect to needed services, even if they are aware of them [18].

One approach to successfully disseminating evidence-based programs for dementia caregivers is to embed them in existing home and community-based services. One such home and community-based service is respite care. Respite care is designed to provide temporary relief to caregivers and can be delivered inside or outside of the home by paid care workers or volunteers [19]. Respite care allows caregivers to have time for their activities while providing a flexible schedule at an affordable cost and may also bolster caregiver resilience [20]. Adult day services (ADS) are an essential form of respite for caregivers of PLWD and also provide various services and social opportunities for older adults living in the community with a range of medical needs [21–23]. In 2020, 5,500 ADS locations provided daily services to 237,400 adults [24]. ADS is the most racially and culturally diverse program compared to other community-based and home-based programs for older adults as well [25].

Although a valued home and community-based service by clients and families, scientific evaluations of ADS dating to the early 1980s have primarily yielded mixed, non-significant results [26, 27]. One approach to strengthening the overall effectiveness of ADS and more fully meeting the needs of users (i.e., older clients and their caregivers) is to integrate existing evidence-based approaches into routine ADS programming. For example, ADS Plus is a caregiver support program embedded in ADS that staff deliver to clients' caregivers. ADS Plus offers 12 months of support to caregivers of clients who attend ADS, which is delivered in person or via telehealth by trained ADS staff. The ADS Plus intervention provides caregivers with education about dementia and other conditions, service referral, self-care strategies, emotional support, and skills training to improve the management of caregiver-identified concerns, including behavioral issues, functional declines, and similar challenges [28]. ADS Plus incorporates successful components of other evidence-based dementia care interventions, including Resources for Enhancing Alzheimer's Caregiver Health II [REACH II; [29]] and Care for Older Persons in the Environment (COPE) [30, 31]. Preliminary and more recent national evaluations found that ADS Plus can reduce caregiver depression, increase the attendance of cognitively impaired clients in ADS settings, and is acceptable to staff [32], among other benefits [33, 34]. 

Although research has emerged documenting the efficacy of several dementia care intervention strategies [35, 36], the implementation potential of these programs is less clear. Alongside determinations of efficacy and effectiveness, understanding the implementation context and potential of dementia caregiver interventions could help to inform and facilitate the dissemination of effective interventions more widely, thus overcoming the common problem of promising dementia care or other scientific innovations “sitting on a shelf” that do not achieve their true public health potential. For these reasons, this paper aims to assess the implementation process and factors that supported and/or hindered the ADS Plus intervention's efficacy, delivery, and uptake.

In a prior qualitative analysis of implementation factors of ADS Plus [32], we utilized an exploratory approach to identify aspects of the ADS Plus intervention that facilitated perceptions of the acceptability and feasibility of the intervention. The relational nature of the intervention, the learning process incorporated as part of ADS Plus training and the intervention itself, and administrative support all emerged as themes that positively influenced perceptions of the acceptability and feasibility of the ADS Plus program. In this follow-up analysis, informed by an implementation framework (see below), we build upon our initial qualitative findings to comprehensively describe the implementation process that contributed to ADS Plus' effectiveness. In this manner, the current study significantly builds upon and extends our prior qualitative research on ADS Plus.

## Methods

The original quantitative outcome analysis of the ADS Plus trial adhered to CONSORT reporting guidelines and is presented elsewhere [34]. As noted above, the current study considers the qualitative data available in the ADS Plus trial to describe the intervention implementation process.

### Implementation framework

**Fig. 1:**
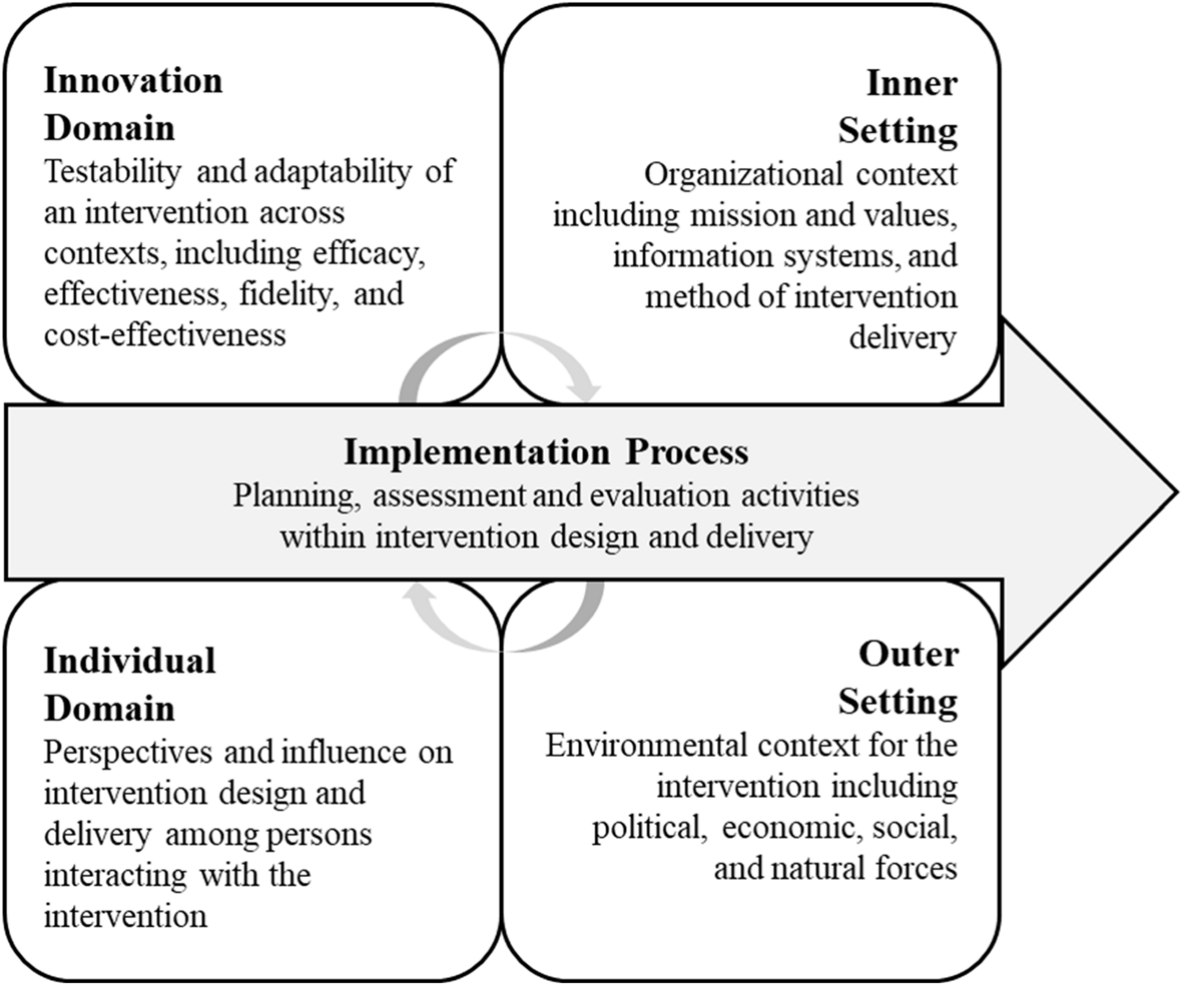
Summary of CFIR domains, adapted from Damschroder and Lowery (2022) [38]

The Consolidated Framework for Implementation Research (CFIR) was developed by the Veterans Affairs (VA) Diabetes Quality Enhancement Research Initiative and provides a conceptual framework for examining factors that influence the development, tailoring, adaptation, and delivery of interventions [37]. We applied CFIR to our examination of the ADS Plus Program to present a comprehensive understanding of implementation factors influencing the intervention’s efficacy, delivery, and uptake [38]. Coupling CFIR with systematic data analysis offers analytic explanatory power in identifying potential associations between implementation factors, processes, and intervention outcomes. CFIR includes five major domains (see Fig. 1). The first domain, Innovation, captures information related to the testability and adaptability of an intervention across contexts, including intervention efficacy, effectiveness, fidelity, and cost-effectiveness. The Outer (i.e., policies, funding landscape) and Inner Setting (i.e., mission and values, information systems, method of intervention delivery) domains capture environmental context and forces that influence intervention implementation. The Individual domain captures the diversity of stakeholder perspectives and the extent to which the involvement shapes intervention design and delivery. The final domain, Implementation Process, examines planning, assessment and evaluation activities within intervention design and delivery.

### Intervention

**Fig. 2:**
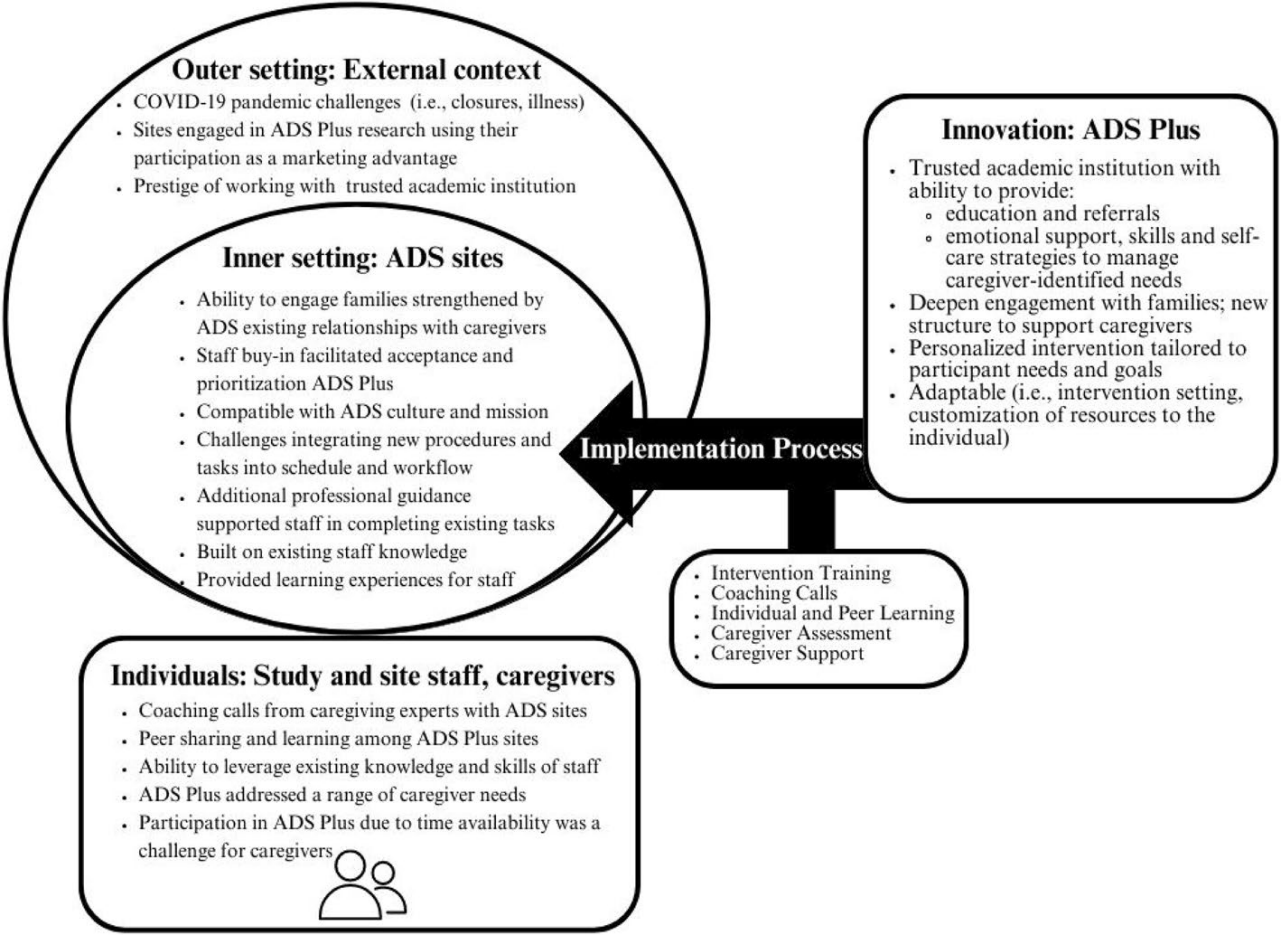
ADS plus program model overview (CFIR), adapted from Damschroder & Lowery (2022) [38]

ADS Plus is designed to provide caregivers with dementia education, referrals, linkages, strategies to address common caregiver-identified challenges (PLWD behavioral symptoms, functional dependence, caregiver concerns such as their own anxiety, planning for the future), stress reduction, and other techniques to care for themselves while offering validation and support [28]. ADS Plus is delivered by staff on site with up to 8 sessions in the first three months and check-in sessions (in person or by telephone) at 4 to 12 months. Each ADS Plus site designated two existing staff members to deliver the ADS Plus intervention. Training involved readings, 16 videos describing intervention components, two 2-h webinars, and monthly coaching calls. Figure 2 illustrates the ADS Plus conceptual model adapted for CFIR. Data in this paper were collected in parallel to the quantitative data of the clinical trial used to evaluate the outcomes of ADS Plus [34]. The detailed protocol of the multi-site hybrid design using mixed methods has been published elsewhere [28]. The study was approved at the Johns Hopkins University (JHU) IRB (Study # 00112546).

### Recruitment

The ADS Plus trial involved 34 ADS sites that volunteered to participate in the study across the United States [28, 34]. Sites were recruited via outreach to interested programs and with guidance from the National Adult Day Service Association and LeadingAge. To be eligible for the trial, sites had to have sufficient size/capacity (≥ 50 clients), ≥ 50% of clients having a dementia diagnosis, and have a sufficient staff-to-client ratio for one staff member to serve as the ADS Plus interventionist and for one to serve as a research coordinator [39].

Participating sites designated a trained research liaison (existing staff member) to inform families at their site of the study via recruitment letters and flyers. Interested family caregivers signed a “consent to be contacted” form to facilitate the research liaison sharing contact information with the study team for screening, consent, and study enrollment. Eligible family caregivers of a PLWD enrolled in a participating ADS site at least one week out of six months, were at least 21 years old, English speaking, had access to a telephone, were willing to participate in four telephone interviews, and were the primary caregiver for a client who had a diagnosis of Alzheimer’s disease or a related dementia.

Of 34 sites, 16 were randomly assigned to provide ADS as usual and the ADS Plus program. Staff at two sites originally invited did not respond to our requests for interviews/scheduling.

### Data collection and analytic procedures

**Fig. 3:**
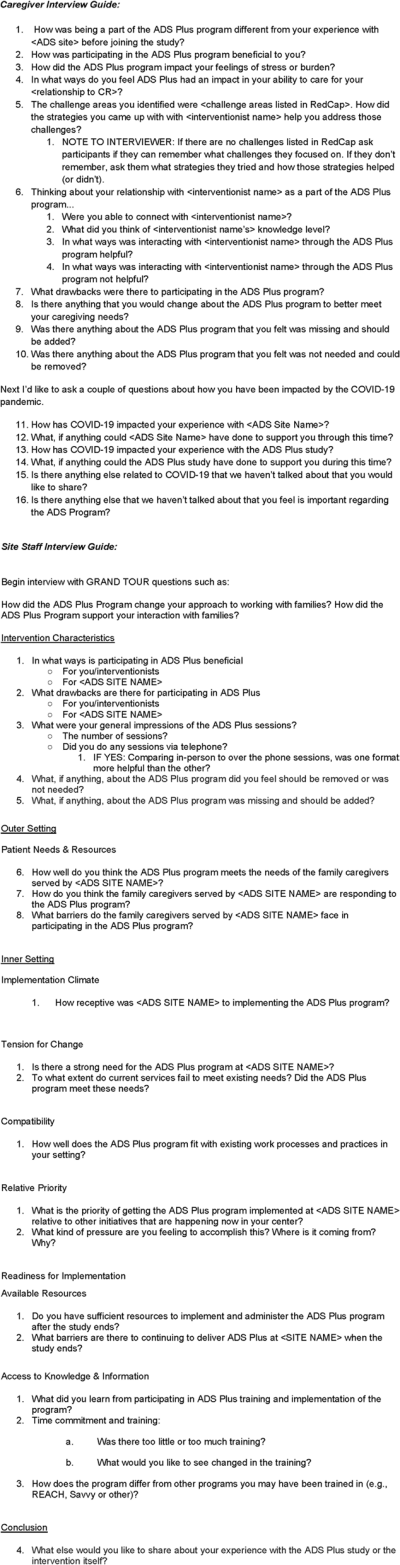
Caregiver and site interview questions used for ADS plus

In parallel with the trial, the University of Minnesota study team conducted semi-structured interviews with 14 ADS Plus site staff and 15 family caregivers at nine of the 16 sites between April 2020 and April 2021, utilizing an interview guide informed by CFIR implementation domains (see Fig. 3). Twenty caregivers who participated in the larger ADS Plus trial were randomly selected for the qualitative interviews. Five caregivers declined to participate. The interviews were transcribed and then analyzed by authors QC, SI, ES, and EA.

**Fig. 4:**
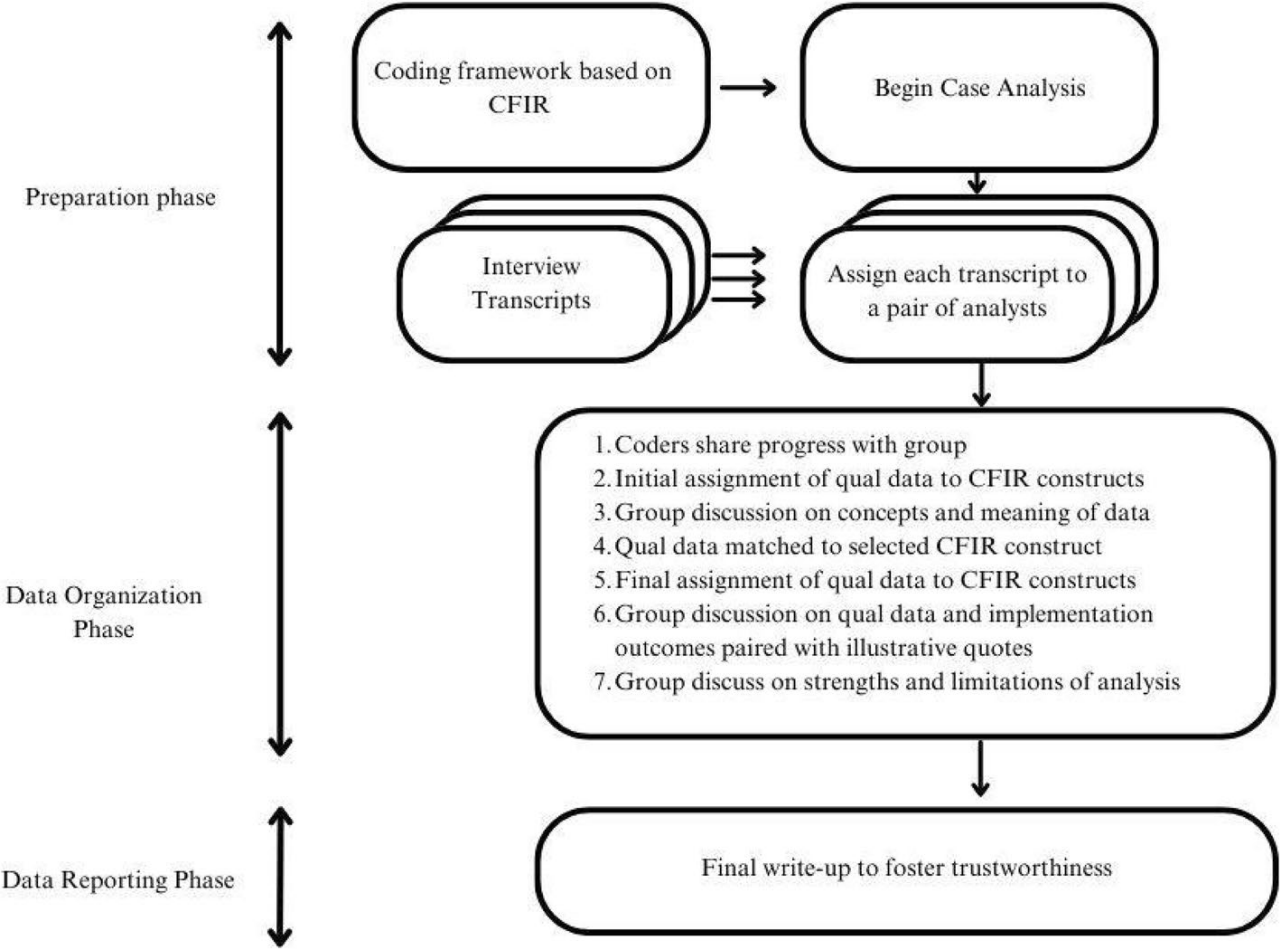
Overview of data analysis (workflow)—adapted from Damschroder & Lowery, 2013 [41] and Assarroudi et. al, 2018 [40]

We conducted a directed qualitative content analysis guided by CFIR [40]. Authors QC, EA, SI, and ES met weekly from October 2022 to May 2023. The authors reviewed data from a randomly selected single-site interview to generate an initial codebook. The site codebook was amended as more data were gathered in subsequent interviews. All interviews were then double-coded in NVivo (version 13). The authors reviewed codes for conceptual clarity and refereed any coding disagreements through a consensus process. Codes were primarily utilized to answer the question: what is happening here? This approach helped to sensitize the analytic team to the data. Blocks of data were then matched to the corresponding five major CFIR domains, and were further refined by QC, EA, SI, and ES. A participant codebook was created using the same process above. Illustrative quotes (see Appendix A) were matched as supporting (or opposing) evidence. See Fig. 4 for an overview of the analytic process. This paper presents our results on relevant major domains and related constructs as per CFIR with supporting evidence.

## Results

Qualitative interview data were available from adult day service intervention site staff (*n* = 14) and family dementia caregivers (*n* = 15) across nine sites randomly assigned to deliver ADS and ADS Plus (see Table 1). Nine caregivers were women, aged 39 to 91 years, and thirteen (86.7%) caregivers reported living with the person who was enrolled in ADS. Eight caregivers (53.3%) were adult children, six were spouses, and one caregiver was of some other relation to the PLWD. Thirteen caregivers (86.7%) reported their race as white. Of the 14 ADS Plus staff members interviewed, 10 acted as interventionists at their ADS site, while the others were research liaisons (*n* = 3) or site administrators (*n* = 1).

**Table 1:** Demographic data of ADS plus caregiver interviewees

Caregiver Characteristics	N (%)
All Caregivers	15 (100.0%)
Gender:	
Women	9 (60.0%)
Relationship:	
Spouse	6 (40.0%)
Adult Child	8 (53.3%)
Other	1 (6.7%)
Race:	
White	13 (86.7%)
Black	1 (6.7%)
Not reported	1 (6.7%)
Ethnicity:	
Non-Hispanic	10 (66.7%)
Lives with ADS Client:	
Yes	13 (86.7%)
	Mean (Standard Deviation)
Age	64.33 (14.7)

Within each CFIR domain, we report on key constructs that provide the greatest insights into mechanisms and processes that underpin the effectiveness and successful delivery of the ADS Plus Program (see Table 2). Our results demonstrate that a detailed implementation protocol adaptable to local needs and coaching by credible intervention designers in an intervention setting with adequate capacity can effectively deliver the intervention. As per CFIR domains, the delivery of ADS Plus was achieved through intervention adaptability, personalization, and structure (innovation); responsiveness of ADS Plus to external changes and intervention marketability (outer domain); presence of aligned goals and familiarity (inner setting); involvement of research staff, connections among practitioners, and meeting caregiver needs (individual domain), and; understanding caregivers needs and addressing staff capacity to take action (implementation process). Globally, our results demonstrate that ADS Plus offers a viable community-based solution for supporting dementia family caregivers.

**Table 2:** Summary of findings

**Innovation Domain**
• Evidence Base: Dementia caregivers reported goal attainment and positively appraised ADS Plus. ADS Plus intervention staff described the intervention’s positive influence on caregivers' confidence • Relative Advantage/Disadvantage: Intervention staff recognize ADS Plus's ability to integrate resources and structure into existing supports, and ADS Plus strengthened caregiver-site connections while addressing dementia caregiver needs • Adaptability: Tailored content and flexibility of ADS Plus delivery methods enabled personalized support to address dementia caregivers’ needs, contributing to the effectiveness of ADS Plus • Innovation Design: Core elements of ADS Plus support goal identification, implementing related action steps, and fostering a sense of empowerment among caregivers. A defined yet flexible program structure drawing on evidence-based practices noted as being an appropriate fit for the setting
**Outer Setting**
• Critical Incidents: COVID-19 disrupted ADS Plus; staffing shortages and reduced in-person services. ADS Plus delivered via telephone to maintain caregiver support • Market Pressure: ADS Plus sites’ commitment to caregivers highlighted. Potential marketing advantage as ADS Plus credibility was associated with a university and evidence-based intervention approach
**Inner Setting**
• Structural Characteristics: Utilizing familiar ADS Service locations with pre-existing caregiver relationships fostered comfort, accessibility, and enhanced usability and acceptability of ADS Plus • Relative Priority: ADS Plus sites prioritized participation, recognizing the intervention’s value and its alignment with their commitment to support impactful caregiving initiatives • Culture and Mission Alignment: Intervention staff noted alignment with their site’s values/mission, expressing enthusiasm for participating in research to support caregivers and enhance care delivery • Compatibility: Intervention staff reported challenges integrating ADS Plus into existing workflows (increased paperwork and work time). Recommendations included streamlined reporting and resources to support activities for dementia caregivers • Access to Knowledge and Information: ADS Plus training provided educational information to enhance staff skills building on their existing knowledge and reintroducing valuable tools. ADS Plus staff reported feeling better equipped to support caregivers
**Individual Domain**
• Implementation Facilitators: ADS Plus staff noted the importance of peer mentorship. Coaching calls with study Principal Investigators described as helpful and increased buy-in for the new intervention among ADS Plus staff • Innovation Deliverers: ADS Plus staff valued hearing from other interventionists to validate their experiences. Interventionists utilized existing relationships, relying on staff familiarity/comfort with care recipients’ history to enhance program delivery • Need: ADS Plus staff offered emotional support, education, self-care strategies, and crisis support. Caregivers valued the practical tools and personalized guidance of knowledgeable interventionists • Opportunity: Caregivers reported difficulty finding time to engage in ADS Plus as intended, citing necessary prioritization of caregiving responsibilities, scheduling and transportation challenges
**Implementation Process**
• Assessing Needs: Interventionists identified dementia caregiver needs through one-on-one sessions and training. Some ADS Plus staff noted feeling rushed and unsure how to proceed after completing training • Assessing Context: Interventionists assessed context through intervention sessions with caregivers, identifying barriers and facilitators to implementation. Peer-sharing and coaching calls provided insights into caregiving challenges and strategies for integrating ADS Plus into sites • Reflecting and Evaluating: ADS Plus staff noted participation in the ADS Plus deepened their sense of importance of data for storytelling and measuring the progress of outcomes • Engaging: ADS sites frequently engaged caregivers through consistent follow-ups and one-on-one interactions. Staff noted consistent follow-up promoted accountability and ADS Plus participation

### Innovation domain

#### Innovation evidence base construct

The Innovation Domain defines innovation as the “thing being implemented" (e.g., the ADS Plus intervention for dementia caregivers) [38]. The use and understanding of lived experiences as evidence of intervention effectiveness provides essential insights into the strength of the core components of an intervention. As available in the ADS Plus qualitative data, the effectiveness of ADS Plus can be defined as the intervention’s ability to provide caregivers with disease education, referral, emotional support, skills, and self-care strategies to manage caregiver-identified challenges. The ADS Plus program achieved this goal for caregivers, and its value was demonstrated among caregivers who reported goal attainment, greater assistance with managing their dementia care circumstances, and positive appraisal of the program. Caregivers described how the structure of ADS Plus, through its provision of individualized support, improved awareness of the importance of self-care and the management of challenges related to dementia care. The intervention ensured that caregivers had regular meeting time to address their issues, received affirmation about the normalcy of their caregiving experience, and provided emotional support that was unavailable through existing connections.

There was the accountability of meeting in person, although that was a little challenging initially. And just having the direct contact in specific areas of concern being addressed. …Like I said, a very structured, very systemized way. And then being able to follow up with any questions, concerns. It just... felt more intimate in the sense that, here I am having all these issues and this is all just for me. That one-on-one was really valuable. (Female, Adult Child)

The ADS program, if nothing else, taught us that it’s okay to be stressed. It’s going to happen. But when it does, it gave us the ability not only to recognize it, but gave us some tools to work through it and also validated that it’s okay to make mistakes. It’s going to happen. It’s okay to take care of yourself because that’s what’s most important, to take care of yourself with stress management or just overall health. (Male, Adult Child)

ADS Plus intervention staff described how the program reached its goal and its influence on caregivers. An interventionist from Site 8 reported how ADS Plus normalized caregiving and increased caregiver confidence about their role. “*I saw that as we were going through the steps that the caregivers became more energized and interested, and developed more confidence in their caregiving role."* Intervention staff also reported that ADS Plus provided space for caregivers to work through challenges, learn, and apply knowledge gained from the program to support issues encountered on the caregiving journey. As noted by one ADS Plus interventionist from Site 5: *“I think them talking it out and hearing it and working through some strategies on some of the needs, they were able to see, “Ah, yes, this can apply to a lot of things.”*

The quotes above emphasize the effectiveness of ADS Plus and reflect the program's core principle (person-centeredness) and treatment components (introducing stress reduction techniques and ways to take care of oneself).

#### Innovation relative advantage/disadvantage

The Relative Advantage/Disadvantage construct is defined by Damschroder et al. (2022) [38] as “the degree to which the innovation is better than other available innovations or current practice.” Overwhelmingly, intervention site staff commented on how ADS Plus added resources and structure to their efforts and improved their ability to relate to caregivers. Overall, ADS Plus provided a structured approach for delivering program content to caregivers and allowed interventionists to explore caregiving concerns in greater detail. ADS Plus site staff identified the value of ADS despite the site having pre-existing resources available for caregivers and that ADS Plus supported deeper connections between the site and families:

My department is also a resource department, so… anyone that was a caregiver-- …could always stop into my department to get different resources. So we have a lot of services going on, a lot of ways that we support, but I do think that this was very-- it was needed because, again, the caregivers wanted to come and sit or talk on the phone. They wanted to have somebody that they could just call on or come in and give resources, or just that support. (Site 2)

I thought that it was unique in the sense of having the day center being an attachment to the added support. It was an added service for families who were enrolled in the day center. So that was a nice draw, and it-- like I said, it just allowed for more of a personal connection between the families who were a part of it. (Site 3)

Some sites preferred ADS Plus compared to other dementia caregiving interventions due to its adaptable nature (e.g., ADS Plus considers objective conditions including person, caregiver, and environmental factors). A staff member from Site 1 noted:

I have been trained to use REACH and I’ve done it a bit, but I far prefer ADS Plus to REACH. What I don’t like about the REACH intervention is how scripted it is. That just doesn’t fit my style. And the prescriptions-- I really love those in ADS Plus. REACH has a similar intervention, but I just think-- it [ADS Plus] just fits me better.

Caregivers pointed out that ADS Plus focused on their needs. In contrast, traditional interventions were for the care recipient: *“So the ADS [Plus Program] for me was more valuable, for me personally, than the [redacted] program*, *needless to say. So the Easter Seal program provided daily service for my mom, so that, I think it's different things* (Male, Adult Child)*.”*

Caregivers frequently reported utilizing more than one type of support, which is a strength of ADS Plus in that it can be integrated with other interventions and supports a center provides. ADS Plus was described as not adequately addressing informational needs in other instances. One caregiver (Female, Spouse) reported: *“I keep combining the two…and it kind of overlapped some. So, it* [the other intervention]* went into more depth than the ADS program.”*

#### Adaptability

The Innovation Adaptability construct describes how an innovation can be adapted to local contexts and needs, tailored, refined, and modified. ADS Plus was designed to be personalized, with tailored content (i.e., “prescriptions”) delivered by the interventionist to each caregiver. One intervention site adapted the mode of delivery of ADS Plus to accommodate a caregiver unable to travel and reported: “*I had a caregiver– I went into her home initially, because she doesn’t drive. So I went to her home for the first three months, and then the rest of it was all phone calls (Site 1).”* At the onset of the COVID-19 pandemic, ADS Plus was modified as a telephone-based intervention instead of relying on in-person delivery, depending on the needs of sites, site staff, and caregivers. This adaptation proved extremely effective and useful for many caregivers who felt isolated due to pandemic restrictions, and many expressed gratitude for having access to the resources and interventionists, despite not being able to visit their ADS program in person. One caregiver reported:

So having to go from where I live to where she was, that was helpful in person, but as I had to do it for so many sessions, it was-- this is probably not even an answer, a positive thing-- that she knew that just became very taxing on me. So when we switched to over the phone, I was like, “Oh my goodness. This is so great. (Female, Adult Child)

A noteworthy adaptation is the tailoring and refinement of resource lists by intervention site staff to ensure that the resources identified were appropriate for caregivers and that amendments to learning content fit the contextual needs of the caregiver.

#### Innovation design

Innovation Design refers to the overall design and presentation of the program [38]. ADS Plus intervention site staff and caregivers appraised the design elements of ADS Plus as favorable overall. ADS Plus provided a structured way for interventionists to work with caregivers to identify their concrete care goals and related actions. A Site 4 staff member reported:

It actually kind of taught us…how to follow in-depth processes for supporting our caregivers. So it kind of provided us with an outline for providing interventions for our caregivers. So a lot of times we just kind of willy-nilly, you know, support our caregivers with as needed things, but I think what she really liked was the fact that it kind of set out goals or outlined things, specific things to work on with our caregivers. So a little bit like what we would call in the social work world action planning where you kind of lay out, like, a little action plan and then proceed with that. So I think ADS Plus did a lot for our program in that sense.

We also examined how sites appraised ADS Plus fit within their respective programmatic contexts. One site described prior experience with evidence-based programming and a focus on increasing service offerings for families, especially for clients with dementia, noting that:

Any time we hear of programs and services that can help us offer more to the community, we’re all about that. We also have the REACH program, which is another evidence-based program specific for dementia, the day center having 99 to 100 percent census of dementia. There’s another reason right there why the ADS Plus study aligned pretty well with what we do*.* (Site 3)

Caregivers pointed out that core elements of ADS Plus, such as information, resources, and contact with intervention staff, supported them in navigating the ADS program and managing their caregiving experiences.

I'm very happy with the program, as you can tell, and it was very concise… it addressed all the issues, and it provided not just conversational guidance or support, but it supported with the physical support, with links and videos and so forth. (Male, Adult Child)

The resources and just the information and knowing that you’re not alone in this, you have access to-- just to information, and, like I said, resources-- to me, that’s invaluable. This was just all new to me, never expected this to happen, and then just to have this resource, I just thought I had won the lottery. I mean, I really did. (Female, Adult Child)

Our qualitative data demonstrates that new innovative programming can be initiated and adapted in adult day service settings when existing or alternative options are perceived as less capable of meeting the needs of adult day service clients and their families. Important considerations for implementing ADS Plus within the Innovation Domain are the effective use of existing organizational infrastructure, a defined program structure that guides staff and clients, personalization of services to meet individual needs, and customizability of support coupled with adult day service staff knowledge and expertise.

### Outer setting

#### Critical incidents

The Outer Setting domain refers to the context and environment in which the ADS Plus intervention exists. Within this domain, we report on Critical Incidents, which examine “large-scale and/or unanticipated events that disrupt implementation and/or delivery of the innovation” [38]. ADS Plus was disrupted by the COVID-19 pandemic, with reductions in site staffing and in-person service delivery being identified as major disruptions. When reflecting on the challenges related to the pandemic, one staff member shared:

The staffing of it, bottom line-- this may be where COVID comes in. We’ve made some significant changes to our staffing here because we had to and to get through it, our period of time here and be good stewards, just going as far as how we were going to end up and making sure that we had who we needed on board and really kind of zeroing in on essential staff. So, when we have no support staff, that makes these kind of above and beyond endeavors that much more challenging. (Site 6)

Some caregivers who could continue the program via telephone expressed appreciation for the staff’s ability to pivot the ADS Plus program amidst the pandemic. One caregiver shared:

I mean, given the support that I had from [my interventionist] with the weekly calls and us just discussing my needs and things that pertained to [Care Recipient], that was-- that just meant so much to me, so I don’t think there was anything else you could-- that the program could’ve done... I’m just grateful that the program continued throughout COVID regardless and that they just didn’t put a halt on the program. (Female, Adult Child)

#### Market pressure construct

The Market Pressure Construct refers to the external pressure ADS facilities may experience when trying to distinguish themselves in the market. Many ADS Plus site staff noted that their participation in ADS Plus held potential as a marketing strategy. Sites noted their participation in the ADS Plus program underscored their commitment to clients and caregivers. One staff member shared:

And if it’s a study that does become an evidence-based study that is then rolled out, there’s marketing to that as well. So just like the REACH program, we are committed to providing that one as an evidence-based program. So, yeah, I believe that ADS Plus would kind of align with what REACH is for us, too. (Site 3)

ADS staff also shared that the prestige of working with Johns Hopkins University was something that caregivers perceived as beneficial. A Site 3 staff member also noted that adopting ADS Plus or a similar intervention would demonstrate agency-level commitment to supporting caregivers. This staff member stated:

A lot of the caregivers were really honored that they got to go through a, you know, a Johns Hopkins, you know, a study like this. So it kind of lends a lot of credit to our agency and what we are providing for our caregivers. (Site 3)

The Outer Setting Domain demonstrates that external forces such as COVID-19 can influence staffing patterns and that the flexible design of ADS Plus supported implementation despite changes in broader environmental conditions. Commitment to supporting older adults and persons with dementia remained throughout COVID-19 among adult day service sites in our study and caregivers also relied on ADS Plus for ongoing support. Additionally, the perceived credibility of ADS Plus was strengthened by the testability of the intervention and potential for becoming an evidence-based program.

### Inner setting

Inner Setting refers to characteristics that contextualize the implementation environment. Within this construct, we report on structural characteristics, culture, compatibility, relative priority, mission alignment, and access to knowledge and information.

#### Structural characteristics

The Structural Characteristics construct describes factors that contribute to the performance of the intervention. ADS sites had pre-existing relationships with caregivers and ADS clients, and services were delivered in a familiar setting, which we found to be a significant contributor toward the usability and acceptability of ADS Plus. The ADS Plus service location did not create additional accessibility issues for caregivers, and familiarity with the service location provided caregivers with a sense of comfort. Both caregivers and staff members pointed out that the embedded nature of ADS Plus fostered relationship building, a vital component as viewed by caregivers:

Well, my experience at [ADS site] is that my [CR] attends the day program, so they know [CR] already, so it was easy to talk to them because they already knew a lot of [CR's] behavior and [CR's] medical history and stuff, versus like a support group where you walk in and they don't know anything, so it was really helpful. (Female, Spouse)

#### Relative priority

The domain of Relative Priority examines the importance of an intervention compared to other initiatives the site is implementing. In our sample, participating sites were all aligned in their commitment to the program while acknowledging that finding the time to participate in such interventions is often difficult. ADS Plus site staff were thoughtful about their commitment and pointed out their belief in the program's value. One staff member shared their perspective on balancing time commitment while wanting to participate in impactful programming, saying:

So there's always tension in adult daycare because when you're just above break even, just above break even, it's hard to find the energy to do additional stuff, but we find it for the right stuff and this is what I consider the right stuff. (Site 7)

#### Culture and mission alignment

The Culture and Mission Alignment constructs represent the feelings staff felt around their shared values, beliefs, and norms that fueled their commitment and purpose as an ADS site and program implementers. Overwhelmingly, staff expressed great interest in implementing ADS Plus to the best of their ability and their openness and optimism about taking on the role of program implementers for the tested intervention. When describing the culture of their site and their philosophy about participating in research, a Site 9 staff member shared: *“The team here prides themselves on being cutting edge and enjoys being a part of any research that would make things easier and more helpful for our caregivers."* Additionally, sites felt that the ADS Plus intervention was well-aligned with the mission of their work and their responsibility to caregivers. Specifically, ADS Plus staff members indicated their strong interest in knowing how best to deliver services to clients and their families.

#### Compatibility

Data matched to the Compatibility Construct identified ADS Plus staff perception on program feasibility. A challenge cited by ADS Plus staff was integrating study activities and program processes into existing workflows. ADS Plus involved additional paperwork and workload, such as meeting requirements for reporting and client assessments. Integrating ADS Plus documentation into existing data systems was a recommendation to ease workload. Staff members also described the dilemma of resource constraints, time, and meeting client needs:

We see the need for it, but the revenue is not always there to support the need…We certainly-- what last I said-- we were doing it informally and fitting things in because we know the value of it, but that just means we work longer hours and don’t get paid for it. It’s always-- we’re always trying to balance what we do and how we do it, but we’re not very good at saying no, so we tend to just do it on our own time. (Site 1)

Although some sites faced challenges integrating ADS Plus into their existing workflows, site staff reported the additional guidance on working with caregivers was a welcome learning experience. Many sites were excited to have this knowledge to inform the way they interact with caregivers in the future.

The ADS Plus framework ensured that individual caregivers were given more support and enhanced the quality of care received. Site 5 shared that ADS Plus gave them more “time to talk to the family one-on-one to find out the best option for them.” In other words, they appreciated the time the intervention set aside to tailor support for each caregiver. Providing the sites with the educational toolkit and space to personalize caregiving resources was one clear advantage of ADS Plus that harmoniously matched existing site goals.

#### Access to knowledge and information

The final construct from the Inner Setting domain was Access to Knowledge and Information, which refers to guidance and training accessible to staff members to implement the program. Many staff noted that the training provided through ADS Plus functioned like continuing education that complemented their previous knowledge. Staff could build on their existing knowledge and were poised to integrate new information. Two site staff reported:

I think it really brushed up on a lot of those skills... That maybe we know, or we've learned, or we've used, but not all the time. It gave us-- it gave me maybe more tools in my toolbox or reminded me of tools that I had used all throughout my social work career. So, it just really made me use some approaches that I had a long time ago or just get more familiar or re-familiar with some of those things. (Site 6)

So for me just going through the training itself and the interventions… the prescriptions and the interventions, it just enhances my ability to provide interventions to caregivers and sort of puts, like, more base knowledge. (Site 4)

Data in the Inner Setting Domain shows that ADS Plus staff perceive their pre-intervention proximity and familiarity with caregivers as a facilitator to program enrollment, rapport building, and understanding of potential needs. The ADS Plus Program aligned with existing goals at sites, added structure to work processes, and staff found that learning from ADS Plus specific training could carry into future interactions with caregivers and improve overall service delivery. Limited financial resources and additional workload were cited as barriers to implementation of ADS Plus and some practical strategies such as efficient use of data systems could address some concerns regarding work demands.

### Individuals

The Individuals Domain refers to the “roles and characteristics of individuals” participating and participating in implementing the innovation [38]. The constructs within this domain pertinent to facilitating the intervention’s success were: Implementation Facilitators, Innovation Deliverers constructs, Need, and Opportunity.

#### Implementation facilitators

The Implementation Facilitators refer to individuals (i.e., study staff and site staff) whose role in the innovation is to provide “subject matter expertise who assist, coach, or support implementation” [38]. One facet of ADS Plus was that the study's Principal Investigators (PIs)-led “coaching calls,” which provided opportunities for sites to provide peer-to-peer mentorship during the program implementation. The direct involvement of the PIs in training allowed sites to receive more robust, expert support, with Site 1 sharing: “*[The PIs] and the others who were on those calls with us were just so supportive. They were great.”* Sites were overwhelmingly positive about the support provided from these calls, with Site 7 further explaining the connection they felt with the project PIs:

[The PI] was great. I mean, I'll tell you too you should send this, and I hope this is reported, but he actually gives a [expletive], and that matters. I mean, I think he was genuinely concerned about the well-being of our program when we first nationwide were shut down. He heard the horror stories of people going through it, and it kind of meant a lot.

#### Innovation deliverers

Another aspect of the Individuals Domain relevant to the facilitation of ADS Plus was the construct of Innovation Deliverers, which refers to individuals “directly or indirectly delivering the innovation” [38]. Site staff members expressed their interest in having space to hear about the experiences of other sites delivering the innovation as it helped to affirm their perceptions and experiences. Sites reported:

It’s always helpful to hear what other interventionists are facing because sometimes, when you’re having some challenges, you’re kind of thinking, Oh, man, am I the only one doing this? Am I doing this right?” And then when you hear someone else say, “Oh, I’m having this problem,” you’re like, “Oh, okay, this makes sense.” So I think they were really important, really beneficial.(Site 2)

I liked when there were those kind of team conference calls. I'm big on hearing how other people are doing with the program or sharing ideas, so I think if it could've been something more like smaller groups or maybe other people that were in the Southwest on a call and then learning what was happening in other parts of the [study]. (Site 8)

Interventionists within ADS Plus utilized their pre-existing knowledge and relationships with families at the site to their advantage. Site staff members were familiar with the issues and history of the care recipients without having to exert extensive energy on building rapport. One caregiver shared:

My [care recipient] attends the day program, so they know her already, so it was easy to talk to them because they already knew a lot of her behavior and her medical history and stuff, versus like a support group where you walk in and they don't know anything, so it was really helpful. The education was great, and the support was great, and it was one-on-one, which was fantastic. (Female, Spouse)

#### Need

The Need construct refers to areas of support among participants addressed by ADS Plus. This construct is a core component of the ADS Plus program, as the intervention is designed as dynamic to address participants' individual needs. ADS Plus addressed a range of caregiver needs by providing services such as emotional support, isolation, education, caregiving and self-care strategies, and resources. Sometimes these services were delivered when a crisis emerged. As described by one caregiver, “*I needed her [interventionist] desperately in a situation that I had no clue how to handle, and she provided every answer. (Female, Adult Child)*” ADS Plus provided caregivers with practical tools and support that helped them to prevent or resolve behaviors. One such tool that the caregivers appreciated was the ability to speak with someone knowledgeable and provide guidance on issues they experienced. One caregiver expressed the range of support provided to address several of their needs:

Oh, it was helpful because it provided the emotional support that sometimes we need when we're dealing with difficult times or difficult behavior. It provided me with the tools that I needed to either prevent the behavior or resolve the behavior, resolutions, and that's something that I felt was very appreciated. It's good to know when these things occur, because I could speak with someone who was knowledgeable and they were able to give you some guidance and some pointers as to how to mitigate the issue or how to prevent the issue from occurring, or how to identify the issue before it becomes a behavior. (Male, Adult Child)

Overwhelmingly, caregivers expressed that having someone to talk to and get information from when they needed help effectively addressed their unmet needs. The relational and personalized nature of ADS Plus resonated with caregivers, with two caregivers reporting:

Definitely...and I am very thankful for that, and for the opportunity to participate in the program. I do not regret having joined the program… you have been an “angel” for me, someone with whom I could talk, open my heart and mind. I otherwise do not have this, I am alone in this country with my mom. (Male, Adult Child)

#### Opportunity

The Opportunity construct refers to the degree to which individuals can fulfill their role in the program. Some caregivers reported facing personal challenges distinct from their caregiving responsibilities that required their attention and acknowledged that these challenges competed with time to be engaged in ADS Plus. It was common among caregivers in our study to report that caregiving responsibilities took precedence, citing difficulty with finding the time to engage in ADS Plus as intended. A few caregivers reported scheduling difficulties and transportation challenges:

Sometimes it was-- sometimes it was difficult to find a time to have an appointment with [interventionist]...But that was because of what was happening in my life, not because of [interventionist]. (Female, Spouse)

If you would ask a recommendation to change things, that would have been personally, for me, just because of the time restrictions and, like I said, it’s a little bit of a distance that I had to go. That’s what-- that was the one thing I would change. (Female, Adult Child)

In the Individual Domain, an important implementation facilitator was engaging study PIs in expert coaching and mentorship, which sites found invaluable for guidance and connection. Innovation Deliverers (ADS Plus sites and staff) leveraged their relationships with families to deliver tailored support. At the same time, caregivers appreciated the program’s ability to meet diverse needs through emotional support, education, and problem-solving strategies. Despite the benefits of ADS Plus, some caregivers faced logistical challenges, such as scheduling and transportation, which limited their engagement in the program.

### Implementation process

Four constructs were critical to understanding the implementation process: Assessing Needs, Assessing Context, Reflecting and Evaluating, and Engaging.

#### Assessing needs

The Assessing Needs construct describes how information was captured about “the priorities, preferences, and needs of the people” involved in ADS Plus [38]. Interventionists learned about “what the caregivers need, what their day-to-day looks like” through one-on-one intervention sessions. Needs included a lack of understanding about dementia, managing behavioral symptoms, and self-care. This was useful for some because their ADS site is “*an agency that helps us to understand how we need to serve families,”* a Site 2 staff member reported. Further, scheduling the intervention sessions was vital in “*identify[ing] issues that were concerning [caregivers] outside of what happens during the adult day time I think was positive. That’s something that didn’t always happen before ADS Plus*” (Site 1). Some interventionists preferred in-person sessions with participants because they could gather more information about caregivers' needs: “*you could kind of see the[ir] reaction[s] and bring things out*” (Site 8). Learning about caregiver needs through staff-client facilitated ADS Plus staff ability to structure services such as mode of delivery or types of services that would be amenable to caregivers. Interventionists also learned about the priorities and needs of caregivers through the training. However, not all interventionists agreed that the training was helpful, such as at Site 2, where an interventionist noted the training “*felt very, very rushed… at the end of it, I wasn’t sure what I was supposed to do*.”

#### Assessing context

The ways individuals assessed context was similar to how they assessed needs: primarily through intervention sessions with caregivers. Accessing Context, or the level to which individuals gathered information to “identify and appraise barriers and facilitators to implementation and delivery of the innovation,” occurred primarily in intervention sessions [38]. Using knowledge gained by other site staff allowed interventionists to identify factors contributing to and detracting from the implementation and delivery of the innovation. Examples cited via peer-sharing included ADS Plus staff having additional insights into the caregiving experience, discussing family engagement opportunities, and learning how to integrate ADS Plus tasks into their workflow. Additionally, ADS Plus site staff mentioned how the coaching calls helped them learn about problems other interventionists had when delivering the innovation at other sites.

#### Reflecting and evaluating

The Reflecting and Evaluating construct is defined as the extent to which individuals “collect and discuss quantitative and qualitative information about the success of implementation.” ADS Plus staff noted the intervention fostered their sense of the importance of data, especially the importance of using data for storytelling and measuring progress to discuss outcomes. Staff at Site 7 began reflecting on the program and were more confident in the innovation work they were doing because:

We started getting calls from families asking us questions they hadn't asked before and some praising the implementation of some of the methodology given through the program that assisted in better outcomes for them.

#### Engaging

The final construct we report on is the Engaging construct, defined as the extent to which individuals “attract and encourage participation in implementation and/or the innovation.” ADS sites tried to engage caregivers in ADS Plus through follow-up before or after enrollment. An interventionist at Site 4 said that since caregivers were already stressed, the interventionists needed to get the participants more involved by “*talking with the caregiver on a consistent basis*.” Similarly, interventionists noted that accountability was essential for encouraging participation in ADS Plus. A Site 2 interventionist noted that caregivers “*really feel that they need that one-on-one with someone more often than just one time out of so many months… That it holds them accountable for either things that they need to do or for self-care.*”

Within the Implementation Domain, our data points out that assessing needs focused on ADS Plus staff understanding caregivers' priorities and challenges through one-on-one sessions was more effective than examples provided in training. ADS Plus staff did find training, coaching calls, and discussions with ADS Plus partner sites from around the country helpful toward identifying barriers and facilitators to implementation. ADS Plus staff noted that these interactions helped them feel validated in their challenges. With implementation occurring in the context of a study, ADS Plus staff did note that the emphasis on data collection facilitated deeper reflection on the importance of data and using data for storytelling. Regarding evaluation, ADS Plus staff noticed that caregivers asked different questions and commented on how new strategies helped them solve issues. ADS Plus staff describe the need for consistent engagement with caregivers and consistent communication fostered accountability to enhance participation in ADS Plus.

Our results show that innovative programming like ADS Plus can be successfully implemented and adapted in adult day service settings when traditional options fall short in meeting client and caregiver needs. Key factors in successful implementation include leveraging organizational infrastructure, providing structured yet customizable programs, and relying on staff expertise to deliver personalized support (Innovation Domain). External factors, such as COVID-19, influenced staffing and operations, but the program’s flexible design and credibility as a potential evidence-based intervention supported its effectiveness during these challenges (Outer Setting Domain). Barriers such as limited financial resources and increased workloads were noted, but strategies like efficient data systems and consistent engagement with caregivers helped mitigate these challenges (Inner Setting Domain).

ADS Plus staff emphasized the value of close caregiver relationships for rapport building and identifying needs, while structured training, coaching, and peer collaboration provided critical implementation guidance (Individual and Implementation Domains). Caregivers appreciated the program’s emotional and practical support but faced logistical obstacles like scheduling and transportation (Individual Domain). ADS Plus engagement was enriched by client-staff, coach-staff, and peer-staff interactions. Data collection in the study context promoted deeper reflection on the program’s impact and the role of outcomes measurement and storytelling Implementation Domain). Overall, consistent communication and engagement were vital for program implementation and caregiver participation (Implementation Domain).

## Discussion

The growing prevalence of dementia in the U.S. underscores a critical public health concern [1, 42, 43]. The extensive and sometimes overwhelming care that families provide, the minimal dementia-specific training they receive, and the lack of available dementia care services have led to a potential crisis in care for PLWD. Numerous dementia care interventions have demonstrated efficacy in improving outcomes for caregivers and PLWD. Still, a significant gap in dementia care science is the lack of attention to implementation considerations when guiding and supporting the dissemination of evidence-based programs that can benefit families in need [44–46].

Utilizing the Consolidated Framework for Implementation Research to frame our content analysis of semi-structured interviews with ADS Plus site staff and caregivers, our results demonstrate that a detailed implementation protocol adaptable to local needs and coaching by credible intervention designers in community-based settings with adequate capacity can result in successful uptake/adoption of the intervention in ADS settings. For example, ADS Plus sites and interventionists adapted mode of delivery (e.g., in-person, remote) for dementia caregivers who did not drive, and during the COVID-19 pandemic, the ADS Plus intervention similarly was adapted for remote delivery that helped to address dementia caregivers' feelings of isolation (i.e., the Adaptability and Innovation Design CFIR constructs). In such instances, essential elements of the ADS Plus intervention (e.g., identification of goals and related actions tailored to need) were maintained.

The application of the CFIR to the ADS Plus qualitative data yielded important insights into the process and success of the intervention’s implementation; in particular, those elements of the intervention that appeared to drive benefits for dementia caregivers, as well as factors that facilitated and hindered intervention implementation. For example, the Innovation and Implementation Process domains revealed that the structure and adaptability of ADS Plus itself facilitated implementation success. ADS Plus was deemed effective on the part of caregivers and ADS staff by providing dementia education, support, skills to manage dementia-related challenges, and self-care approaches to caregivers. In particular, the tailored/individualized nature of the intervention appeared essential to ADS Plus’ effectiveness for dementia caregivers. In addition, ADS Plus was perceived to contribute value to ADS programs’ routine services through its structured model, focus on dementia caregivers’ individual needs, and its adaptability for remote delivery.

As part of the Outer Setting domain, a key critical incident that influenced ADS Plus implementation was the COVID-19 pandemic, where almost all ADS programs participating in the ADS Plus intervention had to physically close due to state health departments’ lockdown orders, resulting in reductions/disruptions in staffing and on-site service delivery. As noted above, the adaptability of ADS Plus to pivot to remote/telehealth delivery while maintaining its structured/tailoring support content was cited by ADS staff and caregivers as a facilitator of implementation. Participating in a national effectiveness trial such as ADS Plus was also noted as a facilitator to marketing participating ADS programs. Qualitative findings aligned with the Inner Setting domain further indicated that existing relationships of ADS programs and staff with dementia caregivers, the structured approach to providing education and skills to dementia caregivers and ADS staff, and engagement with caregivers all facilitated the successful implementation of the intervention and aligned with existing workflows. Time pressures to participate in training and administer ADS Plus were potential Inner Setting barriers, but the close alignment of the intervention with the culture and underlying values of participating ADS programs offset this challenge. Within the Individuals domain of CFIR, the personal involvement and investment of the ADS Plus Principal Investigators in staff training and ongoing coaching calls, the creation of learning “communities” of ADS Plus intervention site staff to learn from each other to help guide implementation efforts, and the individualized and wide-ranging educational and emotional support of the ADS Plus intervention all contributed to implementation adoption and success within ADS programs. Individual factors that inhibited the implementation of ADS Plus were the time demands of caregiving, which posed temporal challenges, and transportation barriers (mainly when the intervention was delivered in person only during its earlier stages of implementation).

One issue often challenging to address when considering the wider dissemination and implementation of dementia care interventions is whether they are “ready” to do so. Within evidence-based practice methodology, interventions are generally considered ready for dissemination and implementation when they demonstrate efficacy through randomized controlled trials and empirical synthesis in meta-analyses [47, 48]. However, as randomized controlled trials and meta-analyses of such studies often prioritize internal validity instead of external validity (generalizability, contextual factors that may moderate intervention effects), implementation scientists have questioned whether relying solely on efficacy results is sufficient. Key considerations such as workflow needs, training, costs, cultural needs, and similar factors often dictate implementation success (e.g., fidelity, appropriateness, acceptability) but are not prioritized in efficacy trials [14, 49].

Utilizing implementation frameworks, such as the Consolidated Framework for Implementation Research, is vital for comprehensively identifying factors that explain the success (or not) of intervention integration, adoption, and embedding into care settings. CFIR was developed to aid in systematically evaluating multidimensional contexts to explicate factors influencing implementation [37]. This study found utility in CFIR through its organization of implementation domains to identify the factors that impacted ADS Plus implementation and informed the open-ended interview questions. Other studies' experiences are consistent with our observations employing CFIR [50, 51]. King et al. [50] found that integrating different constructs with participants (e.g., leaders, stakeholders, or champions) overlapped, which is consistent with our results. Specifically, the Individual Domain of CFIR captures plurality in perspective and experience and facilitates the identification of patterns from different viewpoints. Other studies that utilized the CFIR framework also concur that applying this implementation framework was beneficial in conceptualizing results [52–54].

Our study produced evidence of implementation factors within ADS Plus that can effectively support family dementia caregivers in addressing their needs within ADS settings. As we have emphasized elsewhere [47], the ADS Plus mixed methods design that concurrently collected qualitative and quantitative data on effectiveness and the implementation process offers a flexible approach to understanding how promising dementia care interventions can be embedded/integrated into everyday care contexts. Unlike the more linear model implied by translational science, mixed methods and the joint collection of effectiveness and implementation data [e.g., a "hybrid effectiveness" study; [55, 56]] allow for a more dynamic process. Perhaps most critically, the design utilized here offers insights into aspects of ADS Plus that are essential to driving the intervention's benefits (i.e., mechanisms) and likely must be preserved in later program dissemination and implementation efforts. Specifically, as noted in the qualitative data representing the Innovation Relative Advantage/Disadvantage construct of CFIR, ADS Plus offered added value to programs' existing efforts to engage with and support dementia caregivers via its structured approach to tailor prescriptions that addressed the individualized needs of caregivers. Moreover, the ability of ADS Plus to maintain its essential mechanism of benefit (e.g., structured tailoring of dementia caregiver education and support) when adapting its mode of delivery to address the complexities of COVID-19 and transportation barriers further explains ADS Plus’ potential benefits.

The study was not without limitations. Perspectives from caregivers and staff who were unavailable to participate in the study were not captured, and there was a lack of ethnic/racial representation in our caregiving sample. For ADS Plus site interviews, our study primarily included perspectives from interventionists and only one site administrator. Demographic data for site staff were unavailable, limiting the study’s ability to capture variance in perspectives and experiences (i.e., training pathway and length of time working with adult day service interventions) from site staff at varying levels within an ADS site. Also, as we showed elsewhere [34], caregivers identifying as Hispanic/Latino had lower rates of enrollment and higher rates of attrition than other race/ethnic groups, and staff at sites serving Hispanic/Latino families had difficulties being trained in and offering ADS Plus. Despite these limitations, our robust analysis produced an audit trail to ensure study reproducibility. Future studies can explore variations in experiences among various contextual factors such as urban/rural settings, characteristics of the ADS site itself, staffing levels and overall preparedness/readiness for introducing a new intervention, and characteristics for persons living with dementia and their family caregivers.

The findings extend our prior qualitative analysis of ADS Plus [32] through its focus on implementation processes that led to the successful adoption of the intervention by ADS programs, as well as a focus on those essential elements/mechanisms that appeared to drive benefit for dementia caregivers. This contrasts with our prior qualitative work, which was less focused on the implementation process and more on perceptions of feasibility and acceptability on the part of ADS staff and dementia caregivers. For these reasons, the findings of the current qualitative analysis suggest that the tailored yet structured support offered to dementia caregivers that is core to ADS Plus is amenable to adaptation to ensure that it benefits dementia caregivers receiving the intervention in diverse settings and with various care needs.

## Conclusion

An intervention's efficacy and/or effectiveness is important, but not the only factor contributing to its implementation success. An over-reliance on efficacy testing with a lack of attention to cost, training requirements, workforce capacity, complexity, and similar considerations that are of high relevance to organizations, families, and PLWD who may use and/or adopt an intervention has hindered the dissemination and implementation of innovations. The traditional approach to intervention development, which persists but is linear and often prolonged, has led to gaps in available care and services for people living with dementia, their family caregivers, and the community organizations that support them. Applying innovations in design and methodology that consider efficacy/effectiveness and implementation concerns concurrently can expedite and enrich the dementia care intervention development process. Applying a hybrid effectiveness study approach, as we did for ADS Plus, offers key insights into the implementation context and process of a dementia care intervention. As found in this study, the structured tailoring component of ADS Plus appeared to drive benefits for dementia caregivers, and the flexibility of the ADS Plus intervention in terms of mode of delivery facilitated adoption/implementation on the part of ADS programs. Such findings emphasize that, in identifying mechanisms of benefit, adaptations in the “forms” of an intervention in terms of mode of delivery, length, and cultural content are possible to facilitate improved integration and adoption into community care settings and yet maintain essential benefits. Such results not only position the ADS Plus program for successful dissemination but also offer a roadmap for other dementia care innovations to identify mechanisms of benefit to facilitate implementation in the U.S. and beyond.

## Supplementary Information

Supplementary Material 1.

## Data Availability

The authors have provided supplemental information. These data are not publicly available as they are qualitative, and sharing the data presented in this paper publicly poses threats to confidentiality and privacy to participants. Persons interested in learning more about the data should contact the authors directly.

## Abbreviations

ADS: Adult day services
CFIR: Consolidated Framework for Implementation Research
COPE: Care for Older Persons in the Environment
CR: Care recipient
PLWD: Persons living with dementia
REACH II: Resources for Enhancing Alzheimer's Caregiver Health II
VA: Veterans Affairs
