# Supplementary material for: Understanding the implementation process of the Adult Day Services Plus program
Source: BMC Geriatr. 2025 Feb 13;25:95. doi: 10.1186/s12877-025-05757-4 (PMC11823245; doi:10.1186/s12877-025-05757-4)
Supplement: Supplementary file 1 — Supplementary Material 1. [file 12877_2025_5757_MOESM1_ESM.docx]

**Supplemental Materials**

Appendix A: CFIR Domains and Study Participant Quotes

| **Domain/Construct** | **Definitions** | **Quotes** |
| --- | --- | --- |
| **I. Innovation Domain** | Innovation: The thing being implemented, e.g., a new clinical treatment, educational program, or city service. |  |
| **A. Innovation Source** | The degree to which: The group that developed and/or visibly sponsored use of the innovation is reputable, credible, and/or trustable. | Site 4: A lot of the caregivers were really honored that they got to go through a, you know, a Johns Hopkins, you know, a study like this. So it kind of lends a lot of credit to our agency and what we are providing for our caregivers. |
|  |  | ID 191: I'm happy to have been taken into the study and happy to respond to the interviews, so that's about all I can say about-- Johns Hopkins is ahead of the curve in terms of gathering information on this topic. It's an important topic. |
| **B. Innovation Evidence Base** | The degree to which: The innovation has robust evidence supporting its effectiveness. | ID 149: There was the accountability of meeting in person, although that was a little challenging initially. And just having the direct contact in specific areas of concern being addressed. And, you know, in, like I said, a very structured, very systemized way. And then being able to follow up with any questions, concerns. It just-- and, you know, it just felt more intimate in the sense that: here I am having all these issues and this is all just for me. That one-on-one was really valuable. |
|  |  | ID 209: she would get me information about how to get help, how to get food for my mom… Psychological support, or social behavior, or mental behavior for me. And, and... that is what I liked about her... [she did it] even though she did not have to do any of that for me. We were to simply talk about what I needed and what my mom needed. And yet she would go that extra mile. |
|  |  | Site 3: It definitely allowed more opportunity to get to know the family situation, as well as the caregiver. There was definitely more time to interact with them and kind of dive into what some of their challenge areas were and really work with them on developing strategies, coping skills or whatever the problem was |
|  |  | Site 2: So the caregivers I worked with, they have all said very, very positive things about their experience. They really enjoy-- or really feel that they need that one-on-one with someone for more often than just one time out of so many months. They find that, when they can-- accountability. I heard that word a lot. That it holds them accountable for either things that they need to do or for self-care. So I think that it really benefits when they can meet with someone on a regular basis. |
| **C. Innovation Relative Advantage/Disadvantage** | The degree to which: The innovation is better than other available innovations or current practice. | Site 2: My department is also a resource department, so if anyone that was a caregiver-- they could always stop into my department to get different resources. So we have a lot of services going on, a lot of ways that we support, but I do think that this was very-- it was needed because, again, the caregivers wanted to come and sit or talk on the phone. They wanted to have somebody that they could just call on or come in and give resources, or just that support. Even just to sit and talk to somebody. |
|  |  | ID 267: So the ADS for me was more valuable, for me personally, than the Easter Seal program needless to say. So the Easter Seal program provided daily service for my mom, so that, I think it's different things |
|  |  | Site 1: I can speak to-- I have been trained to use REACH and I’ve done it a bit, but I far prefer ADS Plus to REACH. What I don’t like about the REACH intervention is how scripted it is. That just doesn’t fit my style. And the prescriptions-- I really love those in ADS Plus. REACH has a similar intervention, but I-- ADS Plus, I just think-- it just fits me better. |
| **D. Innovation Adaptability** | The degree to which: The innovation can be modified, tailored, or refined to fit local context or needs. |  |
|  |  | ID 267: it provided the emotional support that sometimes we need when we're dealing with difficult times or difficult behavior. It provided me with the tools that I needed to either prevent the behavior or resolve the behavior, resolutions, and that's something that I felt was very appreciated. It's good to know when these things occur, because I could speak with someone who was knowledgeable and they were able to give you some guidance and some pointers as to how to mitigate the issue or how to prevent the issue from occurring, or how to identify the issue before it becomes a behavior. |
|  |  | ID 149: I just go back to the point where time is-- sometimes freedom is not something we often have. So having to go from where I live to where she was, that was helpful in person, but as I had to do it for so many sessions, it was-- this is probably not even an answer, a positive thing-- that she knew that that just became very taxing on me. So when we switched to over the phone, I was like, Oh my goodness. This is so great. You know? |
| **G. Innovation Design** | The degree to which: The innovation is well designed and packaged, including how it is assembled, bundled, and presented. | Site 4: And it actually kind of taught us more, like, how to follow in-depth processes for supporting our caregivers. So it kind of provided us with an outline for providing interventions for our caregivers. So a lot of times we just kind of willy-nilly, you know, support our caregivers with as needed things, but I think what she really liked was the fact that it kind of set out goals or outlined things, specific things to work on with our caregivers. So a little bit like what we would call in the social work world action planning where you kind of lay out, like, a little action plan and then proceed with that. So I think ADS+ did a lot for our program in that sense. |
|  |  | ID 267: I'm very happy with the program, as you can tell, and it was very concise. In my experience it was very concise, it addressed all the issues, and it provided not just conversational guidance or support, but it supported with the physical support, with links and videos and so forth. |
| **II. Outer Setting Domain** | The setting in which the Inner Setting exists, e.g., hospital system, school district, state. There may be multiple Outer Settings and/or multiple levels within the Outer Setting (e.g., community, system, state). |  |
| **A. Critical Incidents** | The degree to which: Large-scale and/or unanticipated events disrupt implementation and/or delivery of the innovation. | Site 6: The staffing of it, bottom line-- this may be where COVID comes in. We’ve made some significant changes to our staffing here because we had to and to get through it, our period of time here and be good stewards, just going as far as how we were going to end up and making sure that we had who we needed on board and really kind of zeroing in on essential staff. So, when we have no support staff, that makes these kind of above and beyond endeavors that much more challenging. |
|  |  | ID 267: You know I remember something like that at the beginning because there was a delay in us starting the program, and I do remember a conversation with there would be a physical face-to-face meeting, and that would have been done at [the ADS+ site] I believe, or they would send somebody to the house... I remember that, but, no, once we started it was with the COVID and it was already in progress, so it just started, the COVID, so we didn't participate in the program on a face-to-face program, we did all that stuff through the phone. |
| **External Pressure - Market Pressure** | Competing with and/or imitating peer entities drives implementation and/or delivery of the innovation. | Site 3: I believe so, and if it’s a study that does become an evidence-based study that is then rolled out, there’s marketing to that as well. So just like the REACH program, we are committed to providing that one as an evidence-based program. So, yeah, I believe that ADS Plus would kind of align with what REACH is for us, too. |
|  |  |  |
|  |  | Site 4: A lot of the caregivers were really honored that they got to go through a, you know, a Johns Hopkins, you know, a study like this. So it kind of lends a lot of credit to our agency and what we are providing for our caregivers. |
| **III. Inner Setting Domain** | The setting in which the innovation is implemented, e.g., hospital, school, city. There may be multiple Inner Settings and/or multiple levels within the Inner Setting, e.g., unit, classroom, team. |  |
| **A. Structural characteristics (work infrastructure)** | The degree to which: Infrastructure components support functional performance of the Inner Setting. Note: Use this construct to capture themes related to Structural Characteristics that are not included in the subconstructs below (Physical Infrastructure, Information Technology Infrastrecture, Work Infrastructure) | ID 166: Well, my experience at [ADS site] is that my [CR] attends the day program, so they know [CR] already, so it was easy to talk to them because they already knew a lot of [CR's] behavior and [CR's] medical history and stuff, versus like a support group where you walk in and they don't know anything, so it was really helpful. |
|  |  | Site 3: I thought that it was unique in the sense of having the day center being an attachment to the added support. It was an added service for families who were enrolled in the day center. So that was a nice draw, and it-- like I said, it just allowed for more of a personal connection between the families who were a part of it. |
| **D. Culture** | The degree to which: There are shared values, beliefs, and norms across the Inner Setting. Note: Use this construct to capture themes related to Culture that are not included in the subconstructs below. | Site 4: Well, I think at first we were, like, really, really excited about it, you know, because as an Alzheimer's daycare resource center I think we're always excited to be, you know, participating in anything innovative and everything. |
|  |  | Site 2: It was really important for us to participate because we, again, want to make sure that we are doing everything we can do to support the caregivers. And the ADS Plus study is a great way to find that out. |
| **F. Compatibility** | The innovation fits with workflows, systems, and processes. |  |
|  |  | Like I think kind of my only, like my main comment is is there a way so that the program can be implemented intuitively into the systems of what a center is already doing. That would be kind of my primary thing. And a lot of centers may not quite function like we do. Every center's different. But kind of looking at how do we intuitively put in the processes of this program into what a center is already doing, you know. Because we have systems, like, for instance, we have quarterly reports and reassessment reports that we already do. So how do we, you know, how do we, like, sort of optimize what's already in place to have the program be implemented. - Site 4 |
|  |  | Site 3: I thought that it was unique in the sense of having the day center being an attachment to the added support. It was an added service for families who were enrolled in the day center. So that was a nice draw, and it-- like I said, it just allowed for more of a personal connection between the families who were a part of it. |
| **G. Relative Priority** | Implementing and delivering the innovation is important compared to other initiatives. | Site 8: So there's always tension in adult daycare because when you're just above break even, just above break even, it's hard to find the energy to do additional stuff, but we find it for the right stuff and this is what I consider the right stuff. |
|  |  | Site 1: We really took it seriously. We were very proactive in trying to send out information and contacting people individually that we felt could be supported by this. So it was a-- we were really happy to be a part of the initial study, to be able to try this out. |
| **I. Mission Alignment** | The degree to which: Implementing and delivering the innovation is in line with the overarching commitment, purpose, or goals in the Inner Setting. | Site 2: It is very important that [our site] participated in the study. Knowing how to better serve, or knowing what we can do, what [our site] can do to support caregivers, members, and the community-- that’s our mission. So it’s very important. |
|  |  | Site 3: So with that, any time we hear of programs and services that can help us offer more to the community, we’re all about that. We also have REACH program, which is another evidence-based program specific for dementia, the day center having 99 to 100 percent census of dementia. There’s another reason right there why the ADS Plus study aligned pretty well with what we do. |
| **K. Access to Knowledge & Information** | The degree to which: Guidance and/or training is accessible to implement and deliver the innovation. | Site 4: So for me just going through the training itself and the interventions, you know, that like [other staff member] was saying, the prescriptions and the interventions, it just enhances my ability to provide interventions to caregivers and sort of puts, like, more base knowledge I guess. |
|  |  | Site 6: I think it really brushed up on a lot of those skills... That maybe we know, or we've learned, or we've used, but not all the time. It gave us-- it gave me maybe more tools in my toolbox or reminded me of tools that I had used all throughout my social work career, like, oh yeah. So, it just really made me use some approaches that I had a long time ago or just get more familiar or re-familiar with some of those things. |
| **IV. Individuals Domain** | The roles and characteristics of individuals |  |
| **D. Implementation Facilitators** | Individuals with subject matter expertise who assist, coach, or support implementation. | Site 1: Joe and Laura (PI's)and the others who were on those calls with us were just so supportive. They were great. |
|  |  | Site 8: Joseph (PI) was great. I mean, I'll tell you too you should send this, and I hope this is reported, but he actually gives a *%&!, and that matters. I mean, I think he was genuinely concerned about the well-being of our program when we first nationwide were shut down. He heard the horror stories of people going through it, and it kind of meant a lot. |
| **H. Innovation Deliverers** | Individuals who are directly or indirectly delivering the innovation. | Site 2: It’s always helpful to hear what other interventionists are facing because sometimes, when you’re having some challenges, you’re kind of thinking, Oh, man, am I the only one doing this? Am I doing this right? And then when you hear someone else say, Oh, I’m having this problem, you’re like, Oh, okay, this makes sense. So I think they were really important, really beneficial. |
|  |  | ID 166: Well, my experience at [ADS site] is that my [CR] attends the day program, so they know her already, so it was easy to talk to them because they already knew a lot of her behavior and her medical history and stuff, versus like a support group where you walk in and they don't know anything, so it was really helpful. The education was great, and the support was great, and it was one-on-one, which was fantastic. |
|  |  | Site 1: When we were figuring all this out, the coaching calls were helpful. At first, I think we were the only interventionists on some of the first calls, so-- I mean, the only ones that were doing it. There were a lot of people who were still trying to get caregivers enrolled. So it was just the way it was, but it would have been nice initially if there were other interventionists who had been doing it on those first calls. But they were helpful, to be on the phone and talking about just really how the intervention works. |
| *Characteristics Subdomain* |  |  |
| **A. Need** | The degree to which: The individual(s) has deficits related to survival, well-being, or personal fulfillment, which will be addressed by implementation and/or delivery of the innovation. |  |
|  |  | ID 205: I mean, I needed her [interventionist] desperately in a situation that I had no clue how to handle, and she provided every answer, and if she didn’t know-- have an answer, she’d tell me, I’ll call you back tomorrow, and we’ll figure it out. |
|  |  | ID 267: Oh, it was helpful because it provided the emotional support that sometimes we need when we're dealing with difficult times or difficult behavior. It provided me with the tools that I needed to either prevent the behavior or resolve the behavior, resolutions, and that's something that I felt was very appreciated. It's good to know when these things occur, because I could speak with someone who was knowledgeable and they were able to give you some guidance and some pointers as to how to mitigate the issue or how to prevent the issue from occurring, or how to identify the issue before it becomes a behavior. |
| **C. Opportunity** | The degree to which: The individual(s) has availability, scope, and power to fulfill Role. | ID 149: If you would ask a recommendation to change things, that would have been personally, for me, just because of the time restrictions and, like I said, it’s a little bit of a distance that I had to go. That’s what-- that was the one thing I would change. |
|  |  | ID 234: Sometimes it was-- sometimes it was difficult to find a time to have an appointment with [interventionist]...But that was because of what was happening in my life, not because of [interventionist]. |
| **V. Implementation Process** | **The activities and strategies used to implement the innovation.** |  |
| **B. Assessing Needs** | The degree to which individuals: **Collect information about** priorities, preferences, and needs of people. Note: included the subconstructs below (Innovation Deliverers and Innovation Recipients) despite definition. | Site 1: So having a block of time to really sort of identify issues that are concerning them outside of what happens during the adult day time I think was positive. That’s something that didn’t always happen before ADS Plus for some of our members. |
|  |  | Site 2: Everything was very close together because we had those two video sessions and then, in addition to that, there were some online classes that needed to be done. And everything was very rushed. I remember doing it in a couple of months’ time. Excuse me. Maybe two months. Maybe. So everything felt very, very rushed. I kind of feel like I went through all the training but, at the end of it, I wasn’t sure what I was supposed to do. |
|  |  | Site 9: I liked the face-to-face one. Because then you could kind of see the reaction and bring out things. So we tried to have more of those. The phone one, you know, although you designate a specific time for the telephone interaction, I don't think they're the same? You know, and I realize that it's done because of their timing, and it's as their working towards more of the end of the goal. But I think if there could somehow be more of the face-to-face ones, I think those are more beneficial. It gives the caregiver time to be away from all the discussion, because even when you call and you have like a specific time, they still have distractions at home. |
|  |  | Site 6:I think it really gave us the opportunity, or myself the opportunity, to sit down more one on one with some of the families when maybe we don’t necessarily always have the time to do that or-- you know what I mean? |
| **C. Assessing Context** | The degree to which individuals: **Collect information to** identify and appraise barriers and facilitators to implementation and delivery of the innovation. | Site 4: So, you know, and actually health issues. I remember that was one issue with one of our caregivers in completing out her thing was that the caregiver herself was failing and starting to have health issues. So because you're dealing with oftentimes spouses and so they're elderly themselves, so their own health issues, you know. |
|  |  | Site 2: I think they’re really important to have coaching calls. I think that speaking with not only you guys on that-- month-to-month so we can find out if there’s something new that you guys are implementing or maybe there’s a website or a site we can go to to get more resources. It’s always helpful to hear what other interventionists are facing because sometimes, when you’re having some challenges, you’re kind of thinking, Oh, man, am I the only one doing this? Am I doing this right? And then when you hear someone else say, Oh, I’m having this problem, you’re like, Oh, okay, this makes sense. So I think they were really important, really beneficial. |
|  |  | Site 1:I think if we were to-- this was something we were trying to incorporate into what we do, I think there would be a limit as to how many people we could work with at a time just because of all the other things that-- what we do full-time with their care |
| **F. Engaging** | The degree to which individuals: **Attract and encourage participation in implementation and/or the innovation.** Note: Use this construct to capture themes related to Engaging that are not included in the subconstructs below (Innovation Deliverers and Innovation Recipients). | Site 4: And the other barrier would be engaging the caregivers into the program and kind of-- So it takes a lot of work to follow up with the caregivers, to kind of keep them on track. Because as the clinician, you're really the one in charge of following through, right. It can't be held to them, because they're already stressed. So I think that barrier would, you know, kind of be like putting the caregiver to, even if the caregiver said, Oh, my goodness, I really want to learn. This sounds great, getting the caregiver involved and, you know, talking with the caregiver on a consistent basis. |
|  |  | Site 2: So the caregivers I worked with, they have all said very, very positive things about their experience. They really enjoy-- or really feel that they need that one-on-one with someone for more often than just one time out of so many months. They find that, when they can-- accountability. I heard that word a lot. That it holds them accountable for either things that they need to do or for self-care. So I think that it really benefits when they can meet with someone on a regular basis. |
|  |  | Site 1: I had a caregiver-- I went into her home initially, because she doesn’t drive. So I went to her home for the first three months, and then the rest of it was all phone calls. |
| **H. Reflecting and Evaluating** | The degree to which individuals: **Collect and discuss quantitative and qualitative information about the success of implementation.** Note: Use this construct to capture themes related to Reflecting & Evaluating that are not included in the subconstructs below (Implementation and Innovation). |  |
|  |  | Site 1: And I’ve done Powerful Tools for Caregivers and I do like the brevity of some of the ADS Plus-- I mean, I think it just-- maybe the Powerful Tools is a little too scripted for me, as well. This gives you the freedom to sort of present it in your own way, which is much easier for me. It feels like me, as opposed to-- the other one doesn’t feel quite as relationship- based. |
|  |  | Site 4: it actually kind of taught us more, like, how to follow in-depth processes for supporting our caregivers. So it kind of provided us with an outline for providing interventions for our caregivers. So a lot of times we just kind of willy-nilly, you know, support our caregivers with as needed things, but I think what she really liked was the fact that it kind of set out goals or outlined things, specific things to work on with our caregivers. So a little bit like what we would call in the social work world action planning where you kind of lay out, like, a little action plan and then proceed with that. So I think ADS+ did a lot for our program in that sense. -Site 4 |
|  |  | Site 8: We've always strived to be and I felt like we did a decent job of doing it, but we started getting calls from families asking us questions they hadn't asked before and some praising the implementation of some of the methodology given through the program that assisted in better outcomes for them. |
| **Time** |  |  |
|  |  | Site 3: Our challenge is the time commitment every other week and the first eight sessions, especially with the first couple sessions being such a longer session but two- to two-and-a-half-hour sessions. That has been a challenge to add that amount of time on top of the already busy schedules that we were juggling. - Site 3 |
|  |  | Site 3: They both have their benefits. The phone conversations definitely allowed for more flexibility in the schedule |
|  |  | Site 7: we have not had the time and energy to devote to it that we would like to, that we wish we could. |
